# Developing the first pan-Canadian survey on patient engagement in patient safety

**DOI:** 10.1186/s12913-021-07089-6

**Published:** 2021-10-15

**Authors:** Ursulla Aho-glele, Khayreddine Bouabida, Allison Kooijman, Ioana Cristina Popescu, Marie Pascale Pomey, Lisa Hawthornthwaite, Jodi Ploquin, Susan Dunn, Patricia Trbovich, Benoit Tétrault, Maiana Regina Gomes de Sousa, Louise Clément, Nelea Lungu

**Affiliations:** 1 Department of health policy, evaluation and management, University of Montréal / Research Centre of the Hospital Centre of the University of Montreal (CRCHUM), Montreal, Canada; 2 Patient For Patient Safety Canada (PFPSC), Vancouver, Canada; 3 Canadian Patient Safety Institute (CPSI), Ottawa, Canada; 4 Blue Water Health, Sarnia, Canada; 5 Alberta Health Services, Edmonton, Canada; 6 Nova Scotia Health Authority, Halifax, Canada; 7 University of Toronto, Toronto, Canada; 8 Centre Intégré de Sante et de Services Sociaux de Laval, Laval, Canada; 9 Universidade Federal de Goiás, Goiânia, Brazil; 10 Health Standards Organization/ Accreditation Canada, Ottawa, Canada

**Keywords:** Patient engagement, Risk management, Patient safety, Strategies, Mechanism, Tools, Surveys

## Abstract

**Background:**

Patient safety is a worldwide problem, and the patient contribution to mitigate the risk of patient harm is now recognized as a cornerstone to its solution. In order to understand the nature of integrating patients into patient safety and healthcare organizations and to monitor their integration, a Canadian survey tool has been co-constructed by patients, researchers and the Canadian Patient Safety Institute (CPSI). This questionnaire has been adapted from the French version of the patient engagement (PE) in patient safety (PS) questionnaire created for the province of Quebec, Canada.

**Methodology:**

The pan-Canadian PE in PS survey tool was developed in a five-step process: (1) a literature review and revision of the initial tool developed in the province of Quebec; (2) translation of the French questionnaire into an English version tool; (3) creation of a Canadian expert advisory group; (4) adaptation of the English version tool based on feedback from the expert advisory group (assessment and development of the construct’s dimensions, wording assessment and adaptation for pan-Canadian use, technical testing of the online platform for the survey); and (5) pilot testing and pre-validation of the tool before pan-Canadian use.

**Results and conclusion:**

Eight pan-Canadian PE in PS surveys were completed from five Canadian provinces by the expert advisory group and six surveys were completed during the pilot project by participants from different provinces in Canada. This survey tool comprises 5 sections: (1) demographic identification of the participants (Q1 to Q5); (2) general questions (Q6 to Q17); (3) the patient engagement process (experience level of participants and organizational incentives for PE in general) (Q18 to Q33); (4) PE in PS processes, such as current activities, strategies, structures, resources and factors (Q34 to Q67); and (5) the context and impact of PE in PS initiatives in Canadian healthcare organizations (CHOs) (Q68 to Q75), including outcome identification, improvement mechanisms and strategies, evaluation mechanisms, and indicators.

**Supplementary Information:**

The online version contains supplementary material available at 10.1186/s12913-021-07089-6.

## Background

Patient safety^1^ is a worldwide problem. Among the countries in the Organisation of Economic Cooperation and Development (OECD), one in 10 patients are harmed while receiving hospital care [1–4], and nearly 50% of such cases are considered preventable [5]. Worldwide, four out of 10 patients are harmed while receiving health care in a hospital setting, and 80% of such cases could have been prevented [5].

Similar evidence has been found in Canadian hospitals, revealing that “*one in fourteen patients suffer from some form of harm, with a third of such cases being preventable”* [6]. In addition, the latest evidence in Canada reveals that deaths related to such incidents occur every 13 min [7]. A report called “Measuring Patient Harm in Canadian Hospitals” reveals that, in 2014–2015, harm was experienced by patients during one out of every 18 hospital stays, or 138,000 hospitalizations. Of those, 30,000 (or one in five) involved more than one form of harm [8]. Moreover, medical errors in both the acute and home care settings can cost $6800 per patient, resulting in $2.75 billion in additional costs each year in Canada [8]. Estimated costs related to incidents^2^ and accidents^3^ in hospitals represent the costliest form of care, accounting for over $58 billion per year across the country [10]. Similarly, recent evidence demonstrates that 15% of total hospital expenditures and activities in OECD countries is a direct result of adverse events. Estimates show that the total cost of harm in these countries alone amounts to trillions of US dollars every year [4]. One out of every seven Canadian dollars spent on health care is spent treating the effects of patient harm in hospital care [11]. Therefore, it should come as no surprise that investing in the cost of prevention is much lower than the cost of care required due to harm [4].

Today, it is widely recognized that patient engagement (PE)^4^ can help improve outcomes and reduce the burden on health services and on patient safety [12–21]. Indeed, partnering with patients for the sake of their own health and care is known to be a key component for developing the highest quality of healthcare [12, 19, 20, 22–26]. This is why implementing PE strategies offers undeniable value to health care systems by helping reduce by up to 15% the burden of patient harm in hospital care, offering potential savings of billions of dollars each year [4]. So it is imperative for healthcare organisations to be able to assess their PE strategy and implementation status in their organisations to gain a better understanding of their PE performance, specifically regarding risk management (RM), for patient safety (PS) [27].

Patient engagement (PE) has grown in importance as a priority for ensuring quality of care and patient safety (PS) in many Canadian and provincial organizations [28–31]. The knowledge on the best strategies for building a safer health care system grounded in collaboration between patients and healthcare institutions for PS has not yet been properly synthesized [13, 23, 32]. Such strategies address all parts of the system holistically rather than as silos. There is emerging evidence and leading practices on how to implement PE by involving patients in PS [23, 31, 33], but the impacts of such best practices has not been properly researched and demonstrated [12, 19, 20, 23, 34, 35]. In addition, system-wide identification of PS practices is necessary for better-quality and safer healthcare systems [12, 19, 20, 23, 34, 35]. However, existing PE in PS tools mostly identify initiatives at the clinical, organizational or strategic level, without capturing the whole system (see Table 1 for more information: Adapting questions in the tool with the latest evidence from 2017 to 2020).

In this context, the province of Quebec, Canada created a PE in PS questionnaire in 2017. The questionnaire was designed to assess strategies implemented at the level of the health system to integrate PE in PS practices in healthcare organizations [31, 34]. This tool was validated and used from 2017 to 2019 in all healthcare organizations (*n* = 24) in Quebec [35]. Knowing that the questionnaire built in Quebec was created in French, CPSI was asked to adapt the tool in the English language to help healthcare managers assess a system-wide integration of PE in PS practices across the rest of the Canadian country and around the world. The tool incorporated concepts of “Safety I” (situations that can go wrong) and “Safety II” (what goes right and the system’s ability to succeed despite conflicts, uncertainties and risks) [36]. It tracks changes over time based on organizational best practices of PE in PS.

**Fig. 1:**
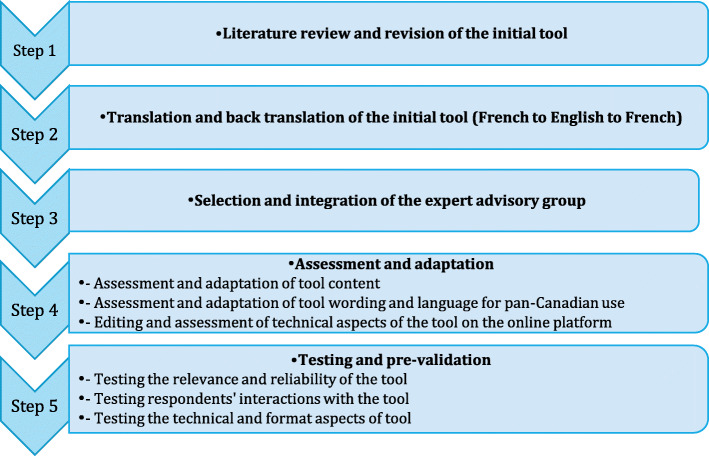
Pan-Canadian survey tool development processes

The purpose of this article is to present the development of a pan-Canadian survey tool to be used by subject matter experts in PE and PS (PS managers, risk managers or a task group, and patient partners) in order to self-assess the nature of PE in PS structures, strategies and factors at the system level in Canadian healthcare organizations (CHOs) and follow up on improvements in these PE in PS strategies over time. A five-step process was used (see Fig. 1 for more information):

- Step 1: Literature review and revision of the initial tool
- Step 2: Translation of the French questionnaire into an English version
- Step 3: Creation of the Canadian expert advisory group
- Step 4: Assessment, adaptation and editing
- Step 5: Pilot testing and pre-validation of the tool

## Methodology

In this section, we present the five different steps taken to develop the Pan-Canadian PE in PS survey tool. Please refer to Fig. 1 for the summary of the different phase to build the survey tool.

### Step 1: literature review and revision of the initial tool

#### Methods

We performed a literature review to complement the work performed developing the French questionnaire and to capture publications published from 2017 to 2019 regarding best practices in PS and PE and/or evaluation tools to survey PE on PS. Health and social sciences databases (PubMed, Medline, Cochrane, CINAHL, EMBASE) were consulted using the keywords “tool” OR “assessment” OR “measurement” OR “questionnaire” AND “patient engagement” OR “patient participation” OR “patient involvement” OR “patient safety.” We also integrated comments and suggested modifications collected during the final validation phase of the French tool, conducted in the province of Quebec, Canada [34] and from a study conducted in France [37] (see Tables 1 and 2 for more information).

**Table 1:** Step 1: Adapting questions in the tool using the latest evidence from 2017 until 2020

Title / Author / Year	Aim	Where	Does the tool focuses on PE in PS?	At which level of the HCO is the tool focussed?
**A 5-facet framework to describe patient engagement in patient safety / Duhn et al./ 2018**	To gain insight into patients’ perspectives about their knowledge, comfort level and behaviours in promoting their safety while receiving health care in a hospital.	Canada	Yes (patient engagement in safety behaviours at the point of care, hospital)	Clinical level
**Public and Patient Engagement Evaluation Tool (PPEET) version 2.0/ McMaster University / 2018**	1. A tool used to assess the organization’s capacity for, and culture of, public and patient engagement; 2. A tool used to obtain participants’ assessments of key features of the engagement activity that they have participated in; 3. A project tool used to assess the planning, execution and impact of the engagement activity after it has been completed.	Canada	No (not focused on patient safety, but rather patient engagement and its institutionalization)	Strategic, organizational or system, and clinical level
**Methods and impact of engagement in research, from theory to practice and back again: early findings from the Patient-Centered Outcomes Research Institute (PCORI) / Forsythe et al./ 2017**	To present PCORI’s evaluation framework for assessing the short- and long-term impacts of engagement; to describe engagement in PCORI projects (types of healthcare providers engaged, when in the research process they are engaged and how they are engaged, contributions of their engagement); and to identify the impacts of engagement on study design, processes, and outcome selection, as reported by both PCORI-funded investigators and patients as well as other stakeholder research partners.	USA	No (not focused on PE in PS but rather on research projects)	Strategic and organizational or system level
**Evaluating patient and public involvement in health research: from theoretical model to practical workshop / Gibson A, et al./ 2017**	To explore the practical utility of the theoretical framework as a tool for mapping and evaluating the experience of patient and public involvement (PPI) in health services research.	England	No (no link to safety)	Organizational or system level

**Table 2:** Step 1: Results from data collected from the study conducted in Quebec and France. Data collected from the study conducted in Quebec and France [38]:

	Quebec	France
**Positive comments**	Relevance of the tool and questions and understanding of the questions: - All respondents mentioned that the tool helped guide actions and could be used as a self-assessment tool for healthcare institutions. The tool is best used by a team of health care professionals (an advisor in PE, RM and or PS and a patient advisor): ° “*The tool helps us really frame our strategies and could be used as a self-assessment tool for healthcare institutions […*] *It will be used for sure.*” Clarity of instructions: - The tool’s instructions were said to be clear and well understood by the team of PE professionals and patients, or PS professionals and patients, or both. ° “You’re asking very pertinent questions, and that helps me.” The choice of having a team answer the questionnaire is very important: ° “It has to be a group that answers the questions. No individual has a comprehensive view of what is going on in the institution.”	
**Limitations**	Limitations related to the questions: - There are many questions about policies and procedures. The absence of policies and procedures does not reflect dedication. Pay attention to the presence of procedures and policies and how they are experienced. - At times it is difficult to answer the questions on the FMG, especially since we do not directly work with them. Limitations related to the structure of the questionnaire: - It is difficult to answer some questions, particularly those related to selecting the percentage of engagement. These sections will need to be worded better instead of using rates (%) of engagement. - Many questions are repeated. - It is easier to complete the questionnaire with the research agent on the other end of the phone.	Bias: Limitations related to the respondents’ points of view: - One of the limitations concerns the points of view expressed. Respondents may have overestimated the actual level of user involvement in their institution or may be unaware of all the initiatives and practices in the many services and branches of the university healthcare centres. Bias: Limitations related to patient groups: - Another limitation of the questionnaire is that it is difficult to differentiate, in the responses, between the involvement of user representatives and that of other patients.
**Deleted questions due to repetition**	In total, 9 questions were deleted: - Questions 67–73 were repetitions so they were deleted (*N* = 7 repeated questions deleted) - 2 questions pertaining to FMGs (family medicine groups) and were also deleted.	

#### Findings

The literature review highlighted four tools for evaluating PE and/or PS at different system levels (see Table 1). Among the four tools identified, none tackled both PE and PS at the organizational or system level. The first tool focused on PE in PS at the clinical level, the second tool focused on PE only at the organizational level. The third and fourth tools focused on PE in research methods and its evaluation.

Suggested modifications from previous studies conducted in Quebec and France were also analyzed and integrated into the assessment of the creation of the pan-Canadian PE in PS survey tool (see Table 2 for detailed information). In summary, participants in the Quebec and French studies reported that they generally had a good understanding of the tool and its questions as well as the instructions and the choice of the participating team in answering the questions:

*“The tool helps us really frame our strategies and could be used as a self-assessment tool for healthcare institutions [ … ] It will be used for sure.”*

*“You're asking very pertinent questions, and that helps me.”*

*“The choice of having a team answer the questionnaire is very important [ … ] It has to be a group that answers the questions. No individual has a comprehensive view of what is going on in the institution.”*

At the end of the evaluation of the Quebec and French studies, nine questions were deleted to avoid repetition and the rewording of certain items, and some questions were scaled to improve understanding. Questions 67–73 were repetitions so they were deleted (*N =* seven repeated questions deleted); two questions pertaining to Family Medicine Groups were also deleted, as they were confusing since not all healthcare organizations did not have Family Medicine Groups and since these questions were specific to the context of the province of Quebec. Other limitations of the previous questionnaire were about the percentages in the questionnaire that were difficult to complete since healthcare organizations did not necessarily know the percentage of engagement of their users in different instances and committees. We thus, modified these sections not to include the percentages, but to just let the participants answer about the degree of involvement of their users (ex: I don’t know, never, sometimes, always, in progress).

Another limitation were about questions on policies and procedures which needed not to just include if the organization had a certain policy or procedure, but also how these policies were integrated and experienced in the organization.

*“The absence of policies and procedures does not reflect dedication. Pay attention to the presence of procedures and policies and how they are experienced.”*

Modifications were brought to these questions (*N* = 25 questions on policies and procedures) and clarification were added to the main question. Here is one example:

Q. **32. The organization has a formal policy on disclosure**
  32a. The disclosure policy is periodically evaluated
  32b. The disclosure policy is evaluated in partnership with patient advisors?

### Step 2: translation of the French tool into an English version

#### Methods

**Fig. 2:**
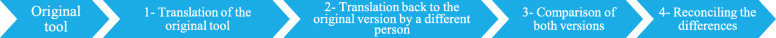
the four steps used in the back translation

The French tool was translated into English and validated following the methodology (back translation) proposed by the Agency for Healthcare Research and Quality [38] and the methodology recommended by the United States Bureau of the Census [39] (see Fig. 2 for more information on the four steps used in the back translation).

#### Findings

The back translation was done in four steps:

1. Translation of the French tool into an English version by the research team.
2. Translation of the English version back into the original language by an external resource
3. Comparing that new translation with the original French tool
4. And, reconciling any meaningful differences between the two versions and bringing about the corrections.

Thus, the tool was translated from the French version used in the Quebec study [34] into an English version, and the English version was translated back into French to confirm the terms used. During the translation and the back-translation, we made sure not to change or modify the meaning or depth of the items in order to not affect the validity of the content.

### Step 3: creation of a Canadian expert advisory group

#### Methods

To adapt the French version, a Canadian expert advisory group was created to adapt the tool to suit a pan-Canadian context, considering the specific features of Canadian health systems. The criteria used to select members were: their knowledge of and expertise in the Canadian health system, patient engagement and patient safety; having already collaborated with CPSI, and their province of origin (for purposes of representation). An initial list of experts was established by CPSI and the research team, emails were sent to the identified individuals, and a final selection was made by the researchers and CPSI using the selection criteria mentioned above.

#### Findings

In total, eight members from five Canadian provinces were recruited and each of them filled out the survey. Thus having eight surveys completed. The eight members are comprised of: 1 academic, 1 patient representative, 4 quality improvement and patient safety specialists, and 2 experts from Accreditation Canada. The research team in charge of developing the study objectives and methodology had five members (three PhD students, one researcher expert in PE, and one advisor from CPSI as a PS and PE projects expert) (see Table 3). In total the working group had 13 members, met 16 times (the research group met with the expert advisory group 4 times and 12 times alone), and on average the meetings lasted 2–3 h.

**Table 3:** Composition of the research team & the expert advisory group for the PE in PS pan-Canadian survey tool

Objective of the research team	- Develop the study objectives and methodology to adapt the pan-Canadian PE in PS tool
**Number of meetings**	12
**How were meetings conducted**	Online meetings through Zoom, in person, and several messages through emails
**Average meeting length**	2–3 h
**Member name**	**Why the individual was chosen for the working group**
Ioana Popescu	Advisor as a PS and PE expert (CPSI commissioned the adaptation, pilot test and first pan-Canadian survey)
Marie-Pascale Pomey	Researcher in PE (created the original Quebec survey)
Khayreddine Bouabida	Student researcher in PE in PS for Canada
Ursulla Aho-Glele	Student researcher in PE in PS in Quebec (created the original Quebec survey)
Maiana R. G. Sousa	Student researcher in PE in PS in Brazil (mainly focused on the literature review to identify PE in PS tools)
**Objective of the expert advisory group**	- Review the tool (dimensions, items, wording, etc.) and support its adaptation and development
**Number of meetings**	4
**How were meetings conducted**	Online meetings through Zoom and email messages
**Average meeting length**	2–3 h
**Organization**	**Why the individual was chosen for the working group**
Bluewater Health	Representative for the Ontario region (quality improvement and patient safety specialist)
Alberta Health Services	Representative for the Western region (quality improvement and patient safety specialist)
Nova Scotia Health Authority	Representative for the Eastern region (quality improvement and patient safety specialist)
Patients for Patient Safety Canada (PFPSC)	Patient representative
CISSS Laval	Representative for the Quebec and Eastern region (quality improvement and patient safety specialist)
University of Toronto, Ontario	Representative for academia
Accreditation Canada	Accreditation body

### Step 4: assessment, adaptation and editing

#### Step 4.1: assessment and adaptation of the tool content

##### Methods

The Canadian expert advisory group reviewed the initial translated English tool for assessment and adaptation purposes. The expert advisory group were also instructed in their revision of the tool to focus on each question and on a more general aspect of it by keeping in mind the flowing interrogations for content and construct validity,^5^ as well as for and reliability^6^:

1. Wording of the questionnaire?
2. Themes covered?
3. What is missing or redundant?
4. Relevance of the questions?
5. Target population (who can answer the questionnaire)?
6. Can the questionnaire be used to assess the reality of patient safety in Canada (so that CPSI and the provinces can use the findings to make better decisions and update the PE in PS guide)?

The goal of the initial tool review and assessment by the expert advisory group was to work as a group using a consensus-building approach to evaluate every single dimension of the initial questionnaire, in order to arrive at a consensus on the content and construct validity as well as on the reliability of the entire tool (i.e. so that all the dimensions used in the Canadian survey would be as relevant, reliable, and exhaustive as possible to fully explore PE in PS in Canada). Note that McDowell and Newell (1987) suggested this method as a way of confirming the content validity of a questionnaire when studying an unknown or new phenomenon in a large organizational or geographic context, such as in our case. McDowell and Newell (1987) suggest that questionnaire dimensions are better developed if they are defined and set up on the basis of the consensus, representativeness, relevance, and exhaustiveness of the constituent items of the concept or the topic to be studied [9, 39].

Based on their knowledge of and expertise in Canadian PE in PS, the expert advisory group assessed all the dimensions of the entire initial tool, item by item. When certain items were unclear, incomplete, or inconsistent, the expert advisory group proposed how they could be corrected or further developed, and then, the expert advisory group would agree or disagree as a group and the consensus was calculated democratically by vote. The highest votes will then be selected to reflect the final decision to be brought as a modification in the survey (to note that we did not have any major disagreements. The expert advisory group came to a consensus through a collaborative strategy). Through the assessment and adaptation process, the expert advisory group was able to create, modify, and adjust certain items to reinforce the initial tool’s (the translated English version) validity and consider the latest trends in PE in PS as practised by CHOs. For example, the initial translated English version tool did not have questions covering the leaders’ own perceptions of change, the methods, and the techniques used by organizations to measure and assess the PE in PS outcomes and change. The initial translated English version tool review sometimes led to minor changes to items or simply adaptations, and at other times it led to the creation of new items based on new trends in PE in PS. For instance, a new item was created on the CHOs’ managers’ perspectives on the impacts of PE in PS and which factors most influence PE in PS, whether by enhancing or limiting PE in PS. Other new items were created in the section on demographic characteristics and information on the respondents’ organizations, such as level of experience with the PE approach, competencies in the field, and level of PE understanding.

##### Findings

The assessment and review of the initial translated English version tool was the key stage in the entire tool development process. The content and construct validity resulted in the creation of a complete adapted version of the tool consisting of 75 items in 5 sections on the following 10 dimensions: (1) demographic characteristics, (2) experience level, (3) incentives, (4) strategies, (5) level of intervention, (6) structure and resources, (7) activities, (8) factors, (9) impacts, and (10) improvements (see Table 4). As per the reliability of the survey, all the experts in the expert advisory group when completing the survey for the second time, were able to give the same answer they gave to the questions with minor differences in their answers to new questions or modified questions.

**Table 4:** Presentation of the dimensions developed in the questionnaire

Dimension Category	Content Description	Questionnaire Sections Item Groups
**Demographic characteristics**	Identify characteristics, e.g. geographic location, facility size, type of care provided, etc., to determine participant profiles (leaders, managers, organizations), using identification characteristics for analysis, interpretation, description purposes.	Section 1 - Demographic Identification questions (organizations and respondents)
**Level of experience**	Determine the level of experience in setting up PE in PS programs (e.g. beginner, in the middle of the process, advanced, etc.) to determine organizational maturity, professional competencies and level of understanding of the PE approach.	Section 1 - Demographic Identification questions
		Sections 2 - General Questions
**Incentives**	Determine the incentive factors for patient engagement (e.g. legislation, outcomes of concern, financial incentives, institutional image, legitimacy, etc.) to determine the motivations of leaders and managers and understand their goals, interests, and perceptions of the patient engagement approach.	Section 3 - Patient Engagement Process (Activities, strategies, structure and resources)
**Strategies (models)**	Identify adopted PE in PS strategies and describe the main practices (intervention model) (e.g. co-design, collaboration, consultation, operational, etc.)	Section 3 - Patient Engagement Process (Activities, strategies, structure and resources)
		Section 4 - Patient Safety Process (Activities, strategies, structure and resources)
**Level of intervention**	Determine the level of PE in PS and the targeted services and components of the organization’s system (clinical, organizational, governance, etc. or any other specific subsystem)	Section 3 - Patient Engagement Process (Activities, strategies, structure and resources)
		Section 4 - Patient Safety Process (Activities, strategies, structure and resources)
**Structure and Resources**	Determine the resources invested in the intervention and implementation of the PE in PS program (e.g. financial, information, structure, material, knowledge, etc.)	Section 3 - Patient Engagement Process (Activities, strategies, structure and resources)
		Section 4 - Patient Safety Process (Activities, strategies, structure and resources)
**Activities (Process)**	Identify the practices to better understand the PE in PS process, implementation dynamic and action mechanisms developed by the organizations (e.g. training, monitoring, communication, etc.)	Section 3 - Patient Engagement Process (Activities, strategies, structure and resources)
		Section 4 - Patient Safety Process (Activities, strategies, structure and resources)
**Factors**	Identify implementation influence factors, i.e. facilitating and limiting factors (e.g. institutional context, support, culture, budget, resistance, etc.) in order to understand the stakes and issues in the implementation process.	Section 3 - Patient Engagement Process (Activities, strategies, structure and resources)
		Section 4 - Patient Safety Process (Activities, strategies, structure and resources), Section 5 - Context and Impact
**Outcomes (Impacts)**	Identify outcomes evaluation and indicator monitoring methods, and explore the perceptions of leaders and managers of the change and the outcomes obtained (e.g. the level of change, the scope, quality, and acceptance of change, avoided costs, etc.)	Section 3 - Patient Engagement Process (Activities, strategies, structure and resources)
		Section 4 - Patient Safety Process (Activities, strategies, structure and resources)
		Section 5 - Context and Impact
**Improvement**	Identify leaders’ and managers’ perspectives on the improvement (e.g. paths of progress, changes, and developments with respect to PE in PS programs for continuous improvement purposes)	Section 5 - Context and Impact

#### Step 4.2: assessment and adaptation of the wording of the tool for pan-Canadian use

##### Methods

In this step, all the members of the expert advisory group carefully examined the wording of every aspect of the tool and suggested improvements when they found inconsistent expressions and vague vocabulary. In addition, two members of Accreditation Canada (AC) performed a careful reading and a deep examination of the tool’s wording, based on their knowledge of and expertise in Canadian healthcare evaluation standards. Here again, a consensus-building approach was adopted to integrate the feedback and the suggestions provided by all the expert advisory group members on the tool’s wording and language.

##### Findings

At the end of this step, our tool was formally defined as pan-Canadian. This step allowed us to adjust its wording to ensure that it would be understood by all CHOs. The expert advisory group’s comments on the adjustments made to the tool’s wording are found in Appendix A. Please refer to Tables 4 and 5 for more information on the changes made to obtain the final version of the pan-Canadian PE in PS survey.

**Table 5:** Step 4: Before and after tool’s adaptation: Layout of the tool

Before adaptation of the tool (Quebec’s initial questionnaire)		After adaptation of the PE in PS pan-Canadian survey tool (after comments from expert advisory group and pilot test)		Description of the adaptation (please refer to Appendix A for more information on comments from the expert advisory group)
Description of Quebec questionnaire sections & dimensions category (Total questions, *N* = 81)		Description of PE in PS pan-Canadian survey tool sections & dimensions category (Total questions, *N* = 75)		
***Section 1. General descriptive questions about the organization*** ***(Questions, N = 14)***	People working in PE in PS	***Section 0. Questions identifying the participants and their organizations (N = 5)***	Type of organization and services provided	**Identify the general characteristics of the participants and organizations:** Switching from focused and specific dimensions (Quebec health system), to more integrated and typical dimensions (Canadian health systems).
	Number of years employed		Type of location (urban, rural) and postal code	
	Type of training received		Job title and department	
	Structure of PE in PS: e.g. department responsible for PE in PS		Years of experience in the position within the organization	
***Section 2. Questions related to PE strategies in general*** ***(Questions, N = 15)***	PE activities	***Section 1. General questions on culture, collaboration tools, and resources or structures contributing to PE in PS (N = 12)***	Existing directorates and departments for implementing and managing PE programs	**Integration and/or modification of additional PE organizational dimensions:** development of new fundamental dimensions for organizing and implementing the PE process (structural, strategic, resources, well-being) as well as new symbolic and complementary dimensions (cultural, communication, etc.).
	Structure and strategies used to engage patients		Mechanisms for collaborating with various departments, committees and community organizations	
	Organization and committees		Budgets and financial investments used to sustain PE integration and incentive factors	
	Training and simulations		Structures, material, and human resources used to engage patients	
	Collaboration with various departments or community organizations		Existing user and patient committees	
	Indicators: implementation, planning and performance		Existing tools and mechanisms for promoting a PE culture	
	Transparency and current policies			
***Section 3. questions related to RM and PS (Questions N = 50)***	PE process and activities	***Section 2. Questions related to the PE process (activities, strategies, structure and resources) at the strategic and organizational level (N = 16)***	PE general strategic plan and PE initiatives and programs	**Integration of additional and/or modification of PE operational and process dimensions:** development of technical dimensions specific to PE processes (training, collaboration, evaluations, incentives, awards, grants, recruitment process, research, conferences, patients as presenters, etc.) necessary to maintaining and monitoring the activities of the PE process.
	Structures used to engage patients		PE training and simulations plans or programs	
	Organization and committee		PE operational planning and process organization	
	Training and simulation		PE indicators and performance measurement (implementation, planning and performance evaluation)	
	Collaboration with various departments or community organizations		PE collaboration mechanisms with various departments, committees or community organizations	
	Indicators (implementation, planning and performance)			
	Transparency and current policies		Development and implementation of PE promotion, transparency and culture policies	
***Section 4. General information on the involvement of the people answering the tool (Questions N = 3)***	Participation of management on PS committees	***Section 3. Questions related patient safety process (activities, strategies, structure and resources at the organizational and clinical level*** ***(N = 34)***	PS general strategic plan and PS initiatives and programs	**Integration of additional and/or modification of organizational and clinical dimensions specific to the PS process:** developing technical dimensions specific to the PS process (training, collaboration, evaluation, monitoring of disclosure, how PE improves PS, etc.) necessary to maintaining and monitoring the activities of the PS process. Developing symbolic and cultural dimensions (transparency and culture policies). Reformulation of questions pertaining to the PS process in order to make a direct link with PE in PS
			Process activities carried out with regard to patient engagement in safety / risk management	
			PS operational planning and process organization	
			PS indicators and performance measurement (implementation, planning and performance evaluation)	
	Additional comments		PS training and simulations plans or programs	
			PS collaboration mechanisms with various departments, committees or community organizations	
			PE promotion, transparency and culture policy development and implementation	
		***Section 4. Context and impact of PE in PS*** ***(N = 8)***	Investment in and improvement of PE in PS in the organization	**Integration of additional and/or modification of contextual dimensions and dimensions of impact and change:** developing dimensions of contributing factors and monitoring and impact evaluation indicators, developing dimensions of improvements in PE in PS, and integrating participants’ and the organizations’ perspectives on the changes.
			Indicators of change and impact of PE on PS	
			Factors influencing PE in PS	
			Documents, guides, processes, and framework that support PE in PS in the organization	
			Additional comments and suggestions	

#### Step 4.3: editing and assessment of technical aspects

##### Methods

This stage was focused on placing the questionnaire on the online platform, testing the technical aspects, and revising the questionnaire before the pilot test. The research team received specific training from the information technology staff at CPSI on how to manage and edit on the online platform. Then the online tool was internally tested by the members of the research team.

##### Findings

At the end of this stage, the questionnaire was set up online, approved by the working group, and considered ready for use in the pilot test. Please refer to Table 5 to see how the questionnaire was developed and adapted from the initial version (French version) to the final version (pan-Canadian version). In addition, please refer to the following link for the pan-Canadian survey, available on the CPSI online platform: https://survey.patientsafetyinstitute.ca/n/zz16p.aspx

### Step 5: pilot testing and pre-validation of the tool

#### Method

In this final phase, we tested and validated the tool among real CHOs. To this end we asked members of the National Health Engagement Network (NHEN), a community of practice, to participate in our pilot test. Once some members had agreed to participate and had given their consent to help test the survey, an email was sent to them explaining the instructions for completing the questionnaire along with the link to the online survey.

#### Findings

The questionnaire was completed in September 2020 by six organizations: 2 in British Columbia, 2 in Ontario, and 2 in Newfoundland. Among these 6 organizations, 3 have a mandate to provide acute care, 2 provide long term care, and 1 is specialized in mental health care. For each organization, the tool was completed simultaneously by a team of three members (one manager in charge of quality and risk management, one person in charge of PE, and one patient advisor). The average time to complete the questionnaire was 52 min, with a maximum of 67 min and a minimum of 27 min.

Once all the responses had been received, the research team exported and analyzed the results on CPSI’s online platform dedicated to the collection and processing of survey data. Based on the pilot test results and participants’ comments, the research team made the necessary adjustments and amendments directly in the tool and emailed it along with the pilot test results to the members of the expert advisory group for final review and approval. Then, all expert advisory group members reviewed the pilot test results, revised the entire tool, approved the changes and adjustments, and gave their final approval. As per the validity and reliability of the survey, the participants were able to complete the survey as per the expert advisory group. We were able to see a consistency in the type of answers giving to a specific question.

Following the pilot test, the tool was considered ready to be used officially in the final validation step: “at the pan-Canadian level.” Table 6 presents the tool items that were modified, adjusted, or adapted based on the pilot test results.

**Table 6:** Step 5: Aspects of the tool that have been modified, adjusted, or adapted based on the pilot test results

Questions / sections modified, or type of comments made		Examples of comments	Action taken to adapt the questionnaire
***Questions modified***	Section (1/A) - General Questions: Q1	Only one person answered this question (5/6 respondents did not answer) *“The first question is important, however it seems to be the weakest and toughest part of the questionnaire (too long and too demanding at the very beginning of the questionnaire). It should be simplified as much as possible!”*	The table was deleted and a “yes or no” question was added, with an opportunity to give an example: 1. Do you have programs, initiatives, activities related to patient engagement in patient safety? Yes No If yes, could you please specify one example: ______________
	Q4		In Question 4 of this questionnaire, we added another example of the definition of a patient advisory council (e.g. *patient engagement committee*)
	Q7	“*Too many to quote them here*”	Question has been simplified to: Give an example if possible
	Q9		In question 9, we deleted “if yes, please specify,” as it was not necessary for this question
	Q12	“*This is not a clear scale. It could be simplified to check boxes*”	We modified Questions 12 & 14 to make it easier to answer by adding check boxes
	Q68	*“This section could be modified into check boxes.”*	In Question 68, we modified the “Comments” section to check boxes and added a “yes or no” question.
Other general comments made by respondents	• The questionnaire could be shortened to 40 min max.		Time to fill out the questionnaire was shortened because questions 1, 12, 14 and 7 were shortened
	• Favouring the check box type of questions rather than the spaces for entering text.		
	• “If yes, please specify” and “please explain your choice” to see if they need to be maintained.		We have deleted the “if yes, please specify” from questions … .

Table 5 shows the final sections (layout) of the pan-Canadian PE in PS tool.

## Discussion

The Pan-Canadian PE in PS survey is an innovative tool to help self-assess the nature of PE in PS structures in CHOs and to monitor changes over time. To our knowledge, no similar tool exists in Canada or elsewhere in the world to identify strategies and initiatives related to PE in PS at a system level. To test its validity, the pan-Canadian PE in PS survey tool was reviewed by an expert advisory group and tested in a pilot test (see Step 5 of the methodology development process).

This article presents a description of the five-step process used to adapt, develop, translate and validate an existing tool on patient engagement (PE) in patient safety (PS), which was co-constructed with patients in the CHOs. The pan-Canadian PE in PS survey tool, intended as a self-assessment tool to be used by subject matter experts in PE and PS who integrate patients, was developed in five steps: (1) a literature review and revision of the French tool; (2) translation of the French questionnaire to English; (3) creation of a working group; (4) assessment, adaptation and editing; and (5) pilot testing and pre-validation of the tool. A preliminary step was also conducted to validate the tool’s content validity (Step 0).

The final version of the PE in PS pan-Canadian survey tool comprises of 75 questions divided into four sections with ten dimensions: Section 0 contains questions for demographic identification of the participants (Q1 to Q5); Section 1 (Q6 to Q17) has general questions to establish participants’ level of experience and organizational incentives for PE in PS; Section 2 (Q18 to Q33) contains questions related to PE processes, such as strategies, activities, structures, resources and factors; Section 3 (Q34 to Q67) has questions on PE in PS processes, such as activities, strategies, structures, resources and factors in place; and Section 4 (Q68 to Q75) contains questions on the context and impact of PE in PS initiatives in the CHOs. More specifically, these questions are focused on outcome identification, improvement mechanisms and strategies, evaluation mechanisms, and indicators.

### The added value of the pan-Canadian PE in PS survey tool

The research team identified six forms of added value provided by the pan-Canadian PE in PS survey tool. First, this is the only self-assessing tool that identifies PE in PS at a system level in healthcare organizations. Based on our research and to our knowledge, no such tool exists at a system level, either in Canada or elsewhere in the world. Second, the tool informs policy and strategic decisions at a national level. At that level, leaders are able to understand the spread and depth of PE in PS across Canada in order to demonstrate what works, which in turn will strengthen the commitment to safe care (by offering evidence-based programs, thereby contributing to one of the five goals of an exceptional healthcare system, as set out in the Canadian Quality and Patient Safety (CQPS) framework [40]). At the provincial level, the tool allows leaders to understand PE in PS in their jurisdictions and how it compares with that of other provinces in order to focus and coordinate their efforts. At the organizational/operational level, the tool allows leaders to understand what works and to implement the practices that can most effectively improve safety. Third, the tool informs practice, identifying the factors, mechanisms, and strategies that effectively improve patient safety through engagement (reduce and prevent harm, reduce the economic burden of patient safety incidents). Fourth, the tool promotes partnering with patients to improve patient safety at the organizational level, and but also to improve care safety at all system levels in Canada. Fifth, the tool and process can be transferred for use in different contexts around the world. Sixth, the tool helps in the CHO accreditation preparation process (e.g. as a survey instrument, a component of the Qmentum^7^ program of Accreditation Canada).

### Strengths of the tool development process

First of all, the tool was adapted from an existing tool tested in a very interesting study conducted in the province of Quebec [34]. This helped create a solid foundation for our tool development process. Second, the creation of the expert advisory group was a tremendous strength in the process used to develop the survey tool. The remarkable expertise and knowledge in PE and PS among the members of the expert advisory group, and the fact that they came from different provinces and worked at different management levels and strategic positions in the CHOs, helped us by enhancing the content validity but also by reinforcing the overall methodology (see Table 3 for more information). And lastly, the involvement of Accreditation Canada (AC) was a great asset, especially in terms of structuring our methodology, defining and selecting the right wording, and connecting with CHOs for the pilot project.

### Limitations

Five limitations of this study were identified during the development of the pan-Canadian PE in PS survey. The first is the fact that an expert translator was not used to translate the initial tool from French to English. However, the research team and the expert advisory group were able to use a back translation method to interpret and adapt the wording to the survey’s pan-Canadian context and review the terms frequently employed by the CHOs in their work environments. The second limitation, which is related to the first, concerns understandings of certain terms. Even though we worked closely with many Canadian experts active in different Canadian heath systems, we believe that this added value resulted in different meanings being ascribed to certain wordings and definitions. For this reason, we incorporated common definitions of certain words that we found differed from one province or CHO to another. We also believe that such discrepancies were few in number. The third limitation concerns the fact that only one patient partner was involved as a member of the expert advisory group. However, the patient partner belongs to a patient organization where the individual has access to an extensive patient partner network. The fourth limitation involves the ability of healthcare professionals to facilitate patient interaction and participation by encouraging patients to ask questions related to patient safety and take an active part in their care and in the healthcare system. Further research will need to identify strategies engage patients in the process of engaging patients to participate in completing surveys in a team setting [41].

The fifth limitation concerns the requirement of having a group of three people (patient safety participant, patient engagement participant, and a resource patient or patient partner) complete the survey together. This could be a major issue for the survey as it becomes more widely used across Canada. While this may add value, it also reduces the chances that the survey will be completed, resulting in a potentially lower response rate. We nevertheless kept this requirement of a group of three people completing the PE in PS survey tool, because of the importance of receiving feedback from the various departments of CHOs.

## Conclusion

The task of developing a new tool by adapting or translating an existing tool into another language and broader context might seem overwhelming. Perhaps the greatest challenge was to produce a tool that is linguistically comprehensible, psychometrically sound, and efficient and effective for use in organizational research settings. This article provides a description of the process used to develop, translate and validate an existing tool for investigating how to engage patients (PE) in patient safety (PS) within Canadian healthcare organizations (CHOs).

The tool is currently being used by healthcare organizations in Quebec and France [37]. A version in Portuguese is currently being prepared to assist healthcare managers in monitoring changes in PE in PS at the system level.

Above all, it must be said that such a tool can meet the WHO’s need to identify and compare PE in PS initiatives around the world. The PE in PS tool that we have created therefore has the potential to help health care organizations identify gaps in their PE in PS as a way to reduce incidents and accidents related to errors by integrating PE into their safety practices. It will also give health care organizations access to a database of recognized international PE in PS practices and strategies, so that they can improve their practices and monitor improvements over time.

The next step in the pan-Canadian PE in PS tool will be use in an official pan-Canadian final validation study in CHOs.

## Supplementary Information

**Additional file 1: APPENDIX A**: Expert advisory group members’ comments after reviewing the initial PE in PS tool.

## Data Availability

All data generated or analysed during this study are included in this article, and the end result is the PE in PS Pan-Canadian survey tool which is available in English: https://survey.patientsafetyinstitute.ca/n/zz16p.aspx

## Abbreviations

CPSI: Canadian Patient Safety Institute
PE: Patient engagement
PS: Patient Safety
RM: Risk Management
CHO: Canadian Healthcare Organizations
OECD: Organization for Economic Co-operation and Development
AC: Accreditation Canada
NHEN: National Health Engagement Network
CQPS: Canadian Quality and Patient Safety
WHO: World Health Organization

## Footnotes

1 Patient safety: The pursuit of the reduction and mitigation of unsafe acts within the health care system, as well as the use of best practices shown to lead to optimal patient outcomes. Patient safety is one of the dimensions of quality. From: https://www.patientsafetyinstitute.ca/en/toolsResources/Patient-Engagement-in-Patient-Safety-Guide/Pages/Glossary-of-Terms.aspx

2 Incidents will be defined here as no-harm incidents: A patient safety incident that reached a patient, but no discernible harm resulted. From: https://www.patientsafetyinstitute.ca/en/toolsResources/Patient-Engagement-in-Patient-Safety-Guide/Pages/Glossary-of-Terms.aspx

3 An “accident” is a situation in which harm was caused and damage occurred [9].

4 The definition this study will abide by is one of *“*Patient Engagement (PE)”, close to the definition of patient partnership, which is defined as patients, families, their representatives, and health professionals working in active partnership, and engaged at various levels across the health care system – clinical level, organizational level and strategic level, − to improve health care and social care as well as patient safety [12].

5 Content validity: This category looks at whether the instrument adequately covers all the content that it should with respect to the variable. In other words, does the instrument cover the entire domain related to the variable, or construct it was designed to measure? Construct validity refers to whether you can draw inferences about test scores related to the concept being studied (Roberta Heale & Alison Twycross. Validity and reliability in quantitative studies, 2015. https://ebn.bmj.com/content/ebnurs/18/3/66.full.pdf).

6 Reliability relates to the consistency of a measure. A participant completing an instrument meant to measure motivation should have approximately the same responses each time the test is completed and among other participants (Roberta Heale & Alison Twycross. Validity and reliability in quantitative studies, 2015. https://ebn.bmj.com/content/ebnurs/18/3/66.full.pdf).

7 Qmentum is a globally developed, locally tailored accreditation program supported by staff and survey experts who have extensive experience in health care at all levels, all around the world. It is designed specifically to help organizations to build resources and capacity, strengthen basic structures and processes related to quality and safety, and help them take part in an accreditation program (https://accreditation.ca/accreditation/qmentum/).
