# Supplementary material for: Developing the first pan-Canadian survey on patient engagement in patient safety
Source: BMC Health Serv Res. 2021 Oct 15;21:1099. doi: 10.1186/s12913-021-07089-6 (PMC8520305; doi:10.1186/s12913-021-07089-6)
Supplement: Supplementary file 1 — Additional file 1: APPENDIX A: Expert advisory group members’ comments after reviewing the initial PE in PS tool. [file 12913_2021_7089_MOESM1_ESM.docx]

**TITLE:** Developing the first pan-Canadian survey on patient engagement in patient safety

**G. APPENDICES**

**APPENDIX A:** **Expert advisory group members’ comments after reviewing the initial PE in PS tool**

| **Section in the survey tool or type of comments** | **Specific comments** |
| --- | --- |
| ***Section 1. Questions identifying the participants and their organizations*** | - In the introduction: asking if they had a PS plan instead of PE plan. |
|  | - Identification of centres #4: the size of these centres? - Do we want to add the first three characters of the postal code to be able to further mine the data? - Identification of individuals #5 and #7: social worker, to add doctors |
|  | - Page 2. The instructions should be made clearer on: using PS or risk management |
|  | - Question #8. Merge Table 8 and the table on Question 5. - Question #4. Which departments have programs, initiatives and activities related to PE in PS, and how many programs, initiatives and activities are carried out in each of these services? (Please complete the table below: This will be a very difficult question to answer in a big organization; suggest that we may want the Accreditation Coordinator to involved in answering this - Types of organizations: Needs to be broader in scope, and make sure it covers the full scope of clients, i.e. home care, rehabilitation, community care, etc. Also need to add organizations that are systems |
| ***Section 2. General question related to the patient engagement process*** | - Question #19. Are training sessions available for patient advisors and PE? Are we thinking of a committee and between professionals and patients? - Section 2. Questions related to patient engagement: This section is well done, and it will be interesting to see the responses, as many questions can be triangulated to the criteria |
| ***Section 3. Questions related patient safety process*** | - Question # 40. Good PS practice, but no connection to PE. It is better to say: do you involve patients in simulation activities? - #64: It is better to say: does senior management take part in the rounds to monitor risks? - Do we have questions related to monitoring of disclosure? Yes, Q46 and Q47 |
|  | - Section 3. Questions related to patient safety: this section is well done, and it will be interesting to see the responses, as many questions can be triangulated to the criteria |
| ***Section 4. Context and impact of patient engagement in patient safety*** | - Section 4. Context and impact: this needs work and should be set up with a Likert scale, like Sections 2 and 3 |
|  | - Additional comments and suggestions for Section 4: - Discussion of perceptions of barriers to PE in PS - Are there any problems with the organizations participating in the survey? |
| **Other general comments on the clarity of the tool and its relevance** | - The questionnaire is clear, and it is an interesting approach to classify questions on PE and on PS. It is not the same person answering these two parts. So this is very worthwhile. It will help the organizations complete the exercise. - The description of the questionnaire was very helpful. - I’m not sure that the icons at the bottom are necessary.   - Relevance of the questions – what do you mean by training in patient engagement? Do you have something specific you think leaders could have to be certified in patient engagement? - The methodology should be clearer; the results may differ if the questionnaire is completed by 3 persons at the same time versus one person at a time. - Add the following words in bold to the methodology part: *When completing the questionnaire, consider activities in the last 12 months* ***in your organization*** |
| **General comments on use of the tool** | - This questionnaire will be useful for organizations. Alignment with AC standards. There are some differences, but a lot of alignment. Highlights where they do well and where they could improve. - The questionnaire is good and will generate a gold mine of information. - The questionnaire was great and easy to follow. - The questionnaire can be used to assess the reality of patient safety in Canada (so that CPSI and the provinces can use the findings to make better decisions and update the guide). - It can be used to assess how patients are engaged in patient safety in organizations…it could identify leading organizations in engagement in risk and safety…it could provide resources to be shared. |
| **General comments on the wording** | - Use “co-designed” as wording in the text - #7: Service department or ward? Add ward? - #13: What would be an example of coordination? Give specific examples. Are there any collaboration activities, e.g. Public Health Agency of Canada, patient bodies, user committees, etc.? - #17: Are there standard forms that the hospital should be using? Do they have a request form? - What do you mean by promotional materials? Give examples of promoting the use of the engagement service: brochures, etc. - #24: What is strategic steering group? - #26: Are patient representatives involved? E.g. patients on the user committee, patients’ association or patient group organization, patients as partners - Add at the beginning a question to have them describe the type of patient group they have. - CEO or director? - 31# and #32: Add “clinical treatment plan.” - #40: Should you leave space for them to indicate other mechanisms for simulation? Do they practice directly on patients, or do they do simulations before? - Specify if (online modules), or formal type of training. Change it to piloting committee? - For consistency, the definition of a patient needs to be consistent with HSO/AC definition if this survey is going to AC clients. - Q 50: What do you mean by “interns”? Do you mean health professionals in training? It needs to be inclusive. - Q7: Does your organization measure patient engagement? Do we mean informal or formal? You need to be specific and ask for examples. - How long have you been working “in the health organization” rather than “in the health sector” |
| **General comments on missing questions** | - Missing questions: does the organisation have awards and incentives for patient engagement? Are there any grants that include plans for PE? What is the process for recruiting, onboarding PE? Conferences, quality events, presence of patient advisors as presenters? Does the organisation use patient’s stories? How? I did not see anything about disclosure. Is there any policy disclosure? - Are they involved in research activities? |
| **Comments on repeated questions** | - Are patients involved in the strategic plan? Make sure the question is not repeated. - #41-43 sound similar….make sure they are different. - #47 and #48, #49 seem redundant with other questions. - Q11: Which department(s) coordinate(s) patient safety? Please check the relevant boxes. How is this different from Question 3? |
| **Other suggestions** | - Put the survey online. - Answer it as an interdisciplinary team, integrating a patient advisor. - Integrate a clinical team, not just at the organizational and strategic level of the organization. - Patients should be actively engaged in this type of activity. - Add: always, sometimes, never. - #22: Instead of circling the correct answer (if on the computer), say make the correct answer bold. - #23: Add, if you answer “Sometimes,” what percentage of the time? - #59-#61: Combine into a multiple choice question or all in one. - Depending on the final version you send out, I would tell people they need to block 45 minutes of their time. - Make it an online survey.  Make the version as easy and clear as possible to fill out, with white space, a larger font, drop-downs etc.  The current format is not as user friendly as it could be. - The scale of measurement is interesting; however, I don’t see the value of “Don’t know” and “In progress.” - How will you perform the aggregate analysis of the information collected? To be explained. Privacy issues? |
